# Supplementary material for: MetaRibo-Seq measures translation in microbiomes
Source: Nat Commun. 2020 Jun 29;11:3268. doi: 10.1038/s41467-020-17081-z (PMC7324362; doi:10.1038/s41467-020-17081-z)
Supplement: Supplementary file 10 — Supplementary Data 7 [file 41467_2020_17081_MOESM10_ESM.zip › File2/Confidence_High_Taxonomy/209499_out.krona.html]

Javascript must be enabled to view this page.

members
magnitude
magnitudeUnassigned
count
unassigned
taxon
rank

209499\_out

2

superkingdom
2
2

74201
1
phylum

class
203494
1

order
48461
1

1647988
1
family

239934
1
genus

1
239935
species

SRS015890\_contig\_number\_20260

1239
1
phylum

class
1
186801

1
186802
order

SRS048411\_contig\_number\_5660
